# Tackling the Waves of COVID-19: A Planning Model for Intrahospital Resource Allocation

**DOI:** 10.3389/frhs.2021.718668

**Published:** 2021-11-16

**Authors:** Felicitas Schmidt, Christian Hauptmann, Walter Kohlenz, Philipp Gasser, Sascha Hartmann, Michael Daunderer, Thomas Weiler, Lorenz Nowak

**Affiliations:** ^1^Asklepios Lung Clinic Munich-Gauting, Gauting, Germany; ^2^Führungsgruppe Katastrophenschutz im Rettungsdienstbereich Fürstenfeldbruck, Fürstenfeldbruck, Germany; ^3^Bio-Inspired Information Processing, Department of Electrical Engineering and Computer Engineering, Technical University of Munich, Munich, Germany; ^4^ÄLRD ZRF-FFB, Fürstenfeldbruck, Germany

**Keywords:** COVID-19, planning model, ICU capacities, health care delivery, pandemic

## Abstract

**Background:** The current pandemic requires hospitals to ensure care not only for the growing number of COVID-19 patients but also regular patients. Hospital resources must be allocated accordingly.

**Objective:** To provide hospitals with a planning model to optimally allocate resources to intensive care units given a certain incidence of COVID-19 cases.

**Methods:** The analysis included 334 cases from four adjacent counties south-west of Munich. From length of stay and type of ward [general ward (NOR), intensive care unit (ICU)] probabilities of case numbers within a hospital at a certain time point were derived. The epidemiological situation was simulated by the effective reproduction number *R*, the infection rates in mid-August 2020 in the counties, and the German hospitalization rate. Simulation results are compared with real data from 2nd and 3rd wave (September 2020–May 2021).

**Results:** With *R* = 2, a hospitalization rate of 17%, mitigation measures implemented on day 9 (i.e., 7-day incidence surpassing 50/100,000), the peak occupancy was reached on day 22 (155.1 beds) for the normal ward and on day 25 (44.9 beds) for the intensive care unit. A higher *R* led to higher occupancy rates. Simulated number of infections and intensive care unit occupancy was concordant in validation with real data obtained from the 2nd and 3rd waves in Germany.

**Conclusion:** Hospitals could expect a peak occupancy of normal ward and intensive care unit within ~5–11 days after infections reached their peak and critical resources could be allocated accordingly. This delay (in particular for the peak of intensive care unit occupancy) might give options for timely preparation of additional intensive care unit resources.

## Background

The first wave of COVID-19 hit Germany relatively weakly in comparison to other countries (1). Potential reasons for this include extensive testing (2), early onset of mitigation measures (1) and the extensive intensive care unit (ICU) infrastructure (3). Additionally, a lot of resources had to be shifted to the care of COVID-19 patients with a subsequent alteration of the health care network. Studies observed not only a decrease in supply in the critical care for, e.g., strokes (4), but also a reduction of demand. For example, fewer emergency room visits were observed in Munich (5) and fewer patients were treated with acute coronary syndromes in German emergency rooms (6). Additionally, waiting time for elective surgery increased and supplies were reduced (5, 7) in order to provide intensive care unit (ICU) capacities for COVID-19 patients. This has led to enhanced economic pressure on hospitals (8). Abating infection rates in June and July 2020 let hospitals return to regular patient care. In August 2020, with eased contact precautions and lifted travel bans for the summer holidays, infection rates were surging again with 15.84 per 100,000 cases in Bavaria [29th of August, 7-day incidence (9)].

Against this backdrop, the question arises when and how hospitals need to react and reallocate their personnel and material capacities to ICU wards and COVID-19 care.

Some of the current models focused on predicting the infection rates in certain populations depending on mitigation measures (10–12). With this estimation, the fraction of infected patients in need for ICU care can be calculated and compared to existing capacities, leading to a valuation of potential capacity related deaths (13). Other models regarding the length of hospital stay concentrated on time-to-death (14). No model so far answers the question when and how hospitals need to react and reallocate further capacities to ICU wards and COVID-19 general wards (NOR) in the light of increasing infection rates. Especially in the absence of an absolute shortage of ICU beds in Germany (15).

The aim of this study was thus to build a model based on the first wave of COVID-19 and validated with real time data from the 2nd and 3rd waves in Germany and to provide possible guidance in the balance act between urgently needed routine patient care and repeatedly increasing (ICU) capacities in case of rising infection rates. This model should answer the following questions:

1. When, after an increase in infections, will bed occupancy rates reach their peaks on normal and ICU wards and consequently, how much time do hospital leaders have to allocate resources accordingly?
2. To what extent will this period be affected by higher infection rates, reflected in *R* and by political mitigation measures?

Finally, the model was validated by comparing the simulated number of infection rates and ICU occupancy with real data obtained during the 2nd and 3rd waves (September 2020–May 2021) in the four counties south-west of Munich (ZRF FFB).

## Methods

### Overview of the Study

Hospital data were collected from five hospitals [Asklepios Lung Clinic Munich-Gauting (abbreviated in the following as Gauting), Starnberg Hospital (Starnberg), Helios Amper Hospital Dachau (Dachau), Fürstenfeldbruck Hospital (Fürstenfeldbruck), and Landsberg Hospital (Landsberg)] in four adjacent counties (Starnberg, Dachau, Fürstenfeldbruck, Landsberg am Lech) south-west of Munich (Supplementary Table 1). Data acquisition occurred in June 2020 and included all cases positively tested for SARS-CoV-2 within these counties between January 28th (when the 1st case was reported in Starnberg, Germany) and June 8th, 2020. The data provided by the hospitals did not contain any patient specific information (e.g., name, birth date). Hospitals provided written consent for the use of their data.

Only retrospective data was used, which was collected and anonymized at the source. Thus, a subsequent assignment of the data file to a specific patient record is impossible. In such cases, the vote of an ethics committee is not necessary according to the guidelines of the responsible ethic committee from the Ludwig-Maximilian University Munich.

We included all provided cases (*N* = 334), categorized by the respective hospital, the length of stay (LOS), and the type of ward the patient was treated in (NOR and ICU; Table 1).

**Table 1:** COVID-19 cases per hospital.

**County**	**Total number of cases**	**Cases per ward**		**Average LOS**			
		**NOR**	**ICU**	**NOR**		**ICU**	
				**M**	**SD**	**M**	**SD**
Gauting	75	74	13	9.7	6.3	15.2	19.2
Starnberg	29	27	7	10.8	6.7	6.7	4.7
Dachau	95	90	37	11.9	9.3	18.4	17.5
Fürstenfeldbruck	96	91	19	9.3	5.9	9.6	8.5
Landsberg	39	34	13	10.0	7.4	9.4	7.0
Total	334	316	89	10.3	7.4	13.8	14.8

*For the normal ward (NOR) and the intensive care unit (ICU) the cases per ward and the average length of stay (LOS) are listed using the mean values (M) and the standard deviation (SD)*.

Day counts for intermediate care (IMC) units were handled as ICU units, due to the similarity of medical treatments and monitoring. Hospitals consented for this approach.

### Input Parameters

Since it is of medical and organizational interest to understand with which probability a new COVID-19 case entering the hospital will stay on the units NOR and ICU for a certain period, the probability distribution as function of time for NOR and ICU is chosen as the main outcome in this analysis.

The probability distribution is defined using the notation of Iverson brackets (16):


(1) $$\begin{array}{r} p_{k}^{w}=\;\frac{1}{N}\overset{N}{\underset{i=1}{\sum }}\left[s_{i}^{w}\geq k\right]\; \end{array}$$

where $p_{k}^{w}\in \left[0.1\right]$ is the probability that a patient stays on day *k* (*k* ∈ {1, 2, 3, …}) on ward *w* (*w* ∈ {*NOR, ICU*}), while *N* is the total number of patients and $s_{i}^{w}$ is the number of days patient *i* stayed on ward *w*. The Iverson bracket converts any logical proposition into a number that is 1 if the proposition is satisfied and 0 otherwise (16).

For both resulting probability distributions exponential and Gaussian fitting was performed.

The probability of occupancy at a given day *k* for a new patient was calculated separately for each of the two wards (NOR; ICU) using Equation (1). The distribution of LOS probability corresponding to the likelihood of occupancy started from initial value close to 1 (0.946) for NOR and 0.267 for ICU and was constantly decreasing. This probability of 0.267 for *k* = 1 also corresponded to the probability that a newly admitted patient needed ICU care for at least 1 day.

**Figure 1:**
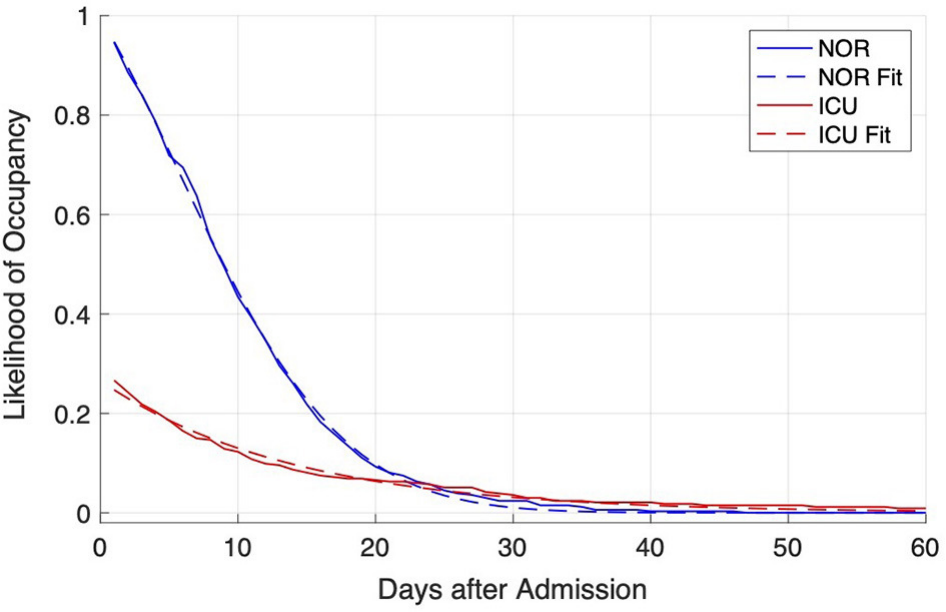
Distribution of likelihood of occupancy at admission of a COVID-19 patient for NOR (blue lines) and ICU (red lines). The distribution was derived from data from the hospitals (solid lines) and fitted with the Matlab fitting app (dashed lines).

The probability of occupancy was fitted with the optimum being achieved by a Gaussian fit for NOR:


(2) $$\begin{array}{r} \tilde{p_{k}^{NOR}}=1.145\cdot e^{-{\left(\frac{k+6.279}{16.74}\right)}^{2}} \end{array}$$

resulting in a root-mean-square error of 0.09887, while for ICU the best accordance was obtained by an exponential fit:


(3) $$\begin{array}{r} \tilde{p_{k}^{ICU}}=0.2655\cdot e^{-0.07145\cdot k} \end{array}$$

resulting in root-mean-square error of 0.005753 (Figure 1).

All calculations and fitting were implemented with Matlab 2020a and its curve fitting application (MathWorks, Inc., code and relevant data provided in repository).

### Model and Assumptions

To demonstrate the applicability of the above obtained probability distribution, we used a simple procedure to gain a notional number of newly infected and hospitalized COVID-19 patients. For this, we started with the incidence in the four adjacent counties similar to the situation mid-August 2020, equal to *I* = 14 (17). Daily number of new infections was calculated using an effective daily growth rate (*g*_*eff*_) derived from the assumed effective reproduction rate (*R*), where new infections were calculated based on infections of last day and effective growth rate of previous day. If the daily growth rate (*g*_*eff*_) is larger than 1, the incidence is increasing, while for values below 1, the incidence is decreasing. The effective reproduction rate (*R*) describes the average number of new infections caused by a single infected individual in the susceptible population. In reference to the Robert-Koch-Institute, we used *R*-values in this model based on the last 8 days that is calculated by dividing the cumulative incidence in the last 4 days by the cumulative new infections in the 4 days before this time interval. The Robert-Koch-Institute assumed that on average 5 days pass between the infection and the onset of the first symptoms (18). However, since transmission might already be possible 2 days before the onset of symptoms, infection of others could already occur just 3 days after personal exposure to COVID-19. The generation time describes the mean time span from the infection of a person to the infection of the subsequent cases infected by them. It corresponds roughly to the serial interval that indicates the mean duration between the onset of the disease in a case and the onset of the disease in its subsequent cases. Robert-Koch-Institute estimated this period of time to be around 4 days due to a relatively high infectiousness at onset and lack of precaution measures if unaware of the disease (18).

Following Wallinga and Lipsitch (19) and an der Heiden and Hamouda (18), the daily growth rate and *R-*value are linked by $g=\sqrt[{4}]{R}$ since two subsiding 4-day intervals are used to calculate the *R*-value (18, 19). Further information on the interdependence of *R*-value and *g* is provided in the online supplement.

The assumptions for the effective reproduction rate (*R*) were based on the observed *R*-values during the 1st wave in Germany (18). For the days prior to first measures, i.e., March 15th 2020, *R*-values of 2 were observed, which reduced to *R*-values of 0.8 within ~9 days after issuing mitigation measures (March 16th 2020) and the lockdown (March 23rd 2020) (18).

**Figure 2:**
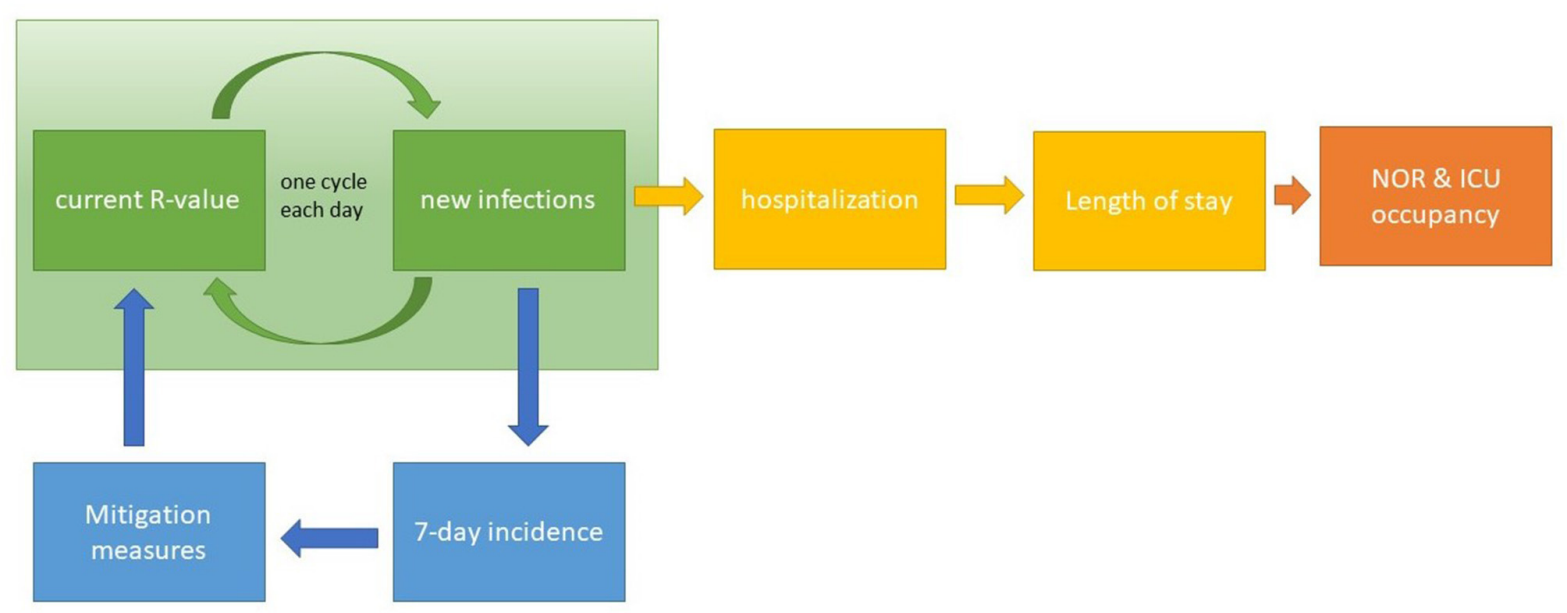
Schematic illustration of the model. The number of new infections is calculated for each day based on the respective *R*-value (green section) and is used to derive the number of new hospitalizations. Length of stay and the number of hospitalizations allow for the estimation of the occupancy of NOR and ICU (section to the right). Based on the 7-day incidence value derived from the number of new infections, mitigation measures are controlled, which influence the current *R*-value (section to the bottom).

The incidence per 100,000 population ($i_{100,000}^{7\;days}$) was calculated within the last 7 days. It was assumed that the government would adopt measures as soon as the 7-day incidence reached $i_{100,000}^{7\;days}$ = 50. This assumption is based on observed 7-day incidences prior to the start of measures during 1st wave in Germany (18), also during the 2nd and 3rd waves the 7-day incidence of 50 was an important threshold to trigger or release measures. These actions could achieve a reduction of the *R*-value and the effective growth rate. The reduction was assumed to occur in nine steps, i.e., within 9 days, to reflect some inertia and delay and was limited to minimal value of *R* called *R*_low_, which is in line with the observed *R*-values during 1st wave (18). Measures were in place for at least *M* days and until $i_{100,000}^{7\;days}$ was below $i_{100,000}^{7\;days}$. The number of new hospitalizations, i.e., new admissions *A*, was deducted from the resulting number of new infections per day (*I*) at a certain hospitalization rate (*H*), namely *A* = *H*·*I*. The hospitalization rate was assumed to be equal to *H* = 17% (20). Using the above obtained probability distributions for NOR and ICU, the resulting occupancy *O*^*NOR*^, *O*^*ICU*^ for NOR and ICU were calculated, respectively. Schematically, this model is reflected in Figure 2. The number of new infections is iteratively calculated using the current *R*-value (green). From the number of new infections, the 7-days incidence can be derived, which in turn constitutes the information criterion for the activation of mitigation measures (blue). These measures affect the current *R*-value. Additionally, from the new infections, the number of new hospitalizations can be calculated. Combined with the information about the length of stay, the NOR and ICU occupancy rates are derived.

## Results

### Characteristics of Length of Stay Data

Data from *N* = 334 patients was collected from the five major hospitals in adjacent counties south-west of Munich comprising a population of 629,295 inhabitants and 1,591 hospital beds (including a maximum number of 126 ICU beds; Supplementary Table 1). The individual hospital case counts ranged from *N* = 29 cases in the Starnberg hospital to *N* = 96 cases in Fürstenfeldbruck.

Sorted according to the type of ward, *N* = 316 patients stayed on average 10.3 (SD 7.4) days on a general ward and on average 13.8 (SD 14.8) days on ICU (*N* = 89 patients). The average length of stay on a general ward differed from 9.3 (SD 5.9) days in Fürstenfeldbruck to 11.9 (SD 9.3) days in Dachau. The highest number of ICU admissions were observed in Dachau with *N* = 37 (Table 1).

### Model Results

**Figure 3:**
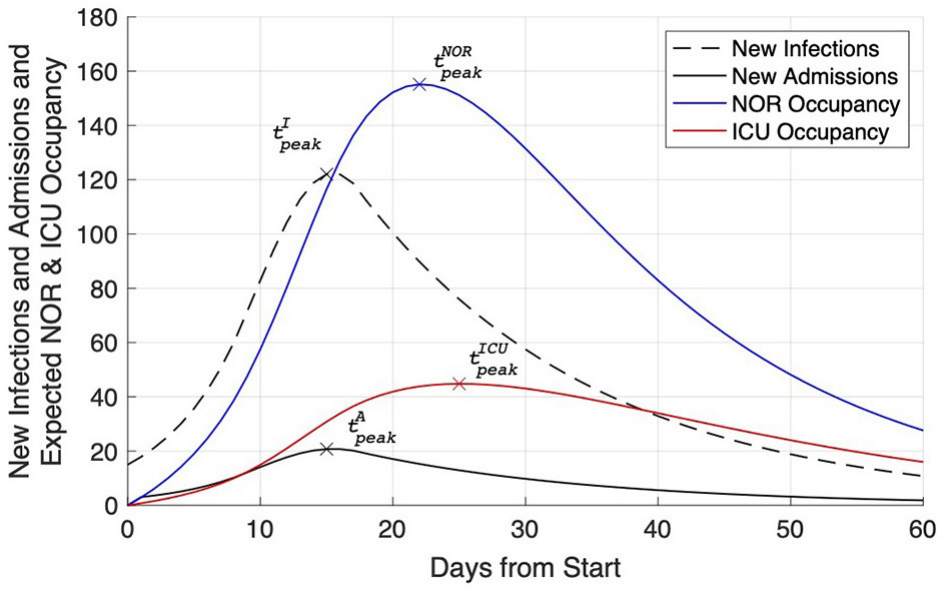
Expected NOR (blue line) and ICU (red line) occupancy due to a notional number of newly admitted Covid-19 patients. Additionally, the number of new infections (dashed black line) and the derived number of new hospital admissions (solid black line) are plotted. For each line the time of peak is indicated.

In a second step, we modeled this LOS against the backdrop of the epidemiological situation. With a reasonable incidence of SARS-CoV-2 in the area, namely *I* = 14, and the hospitalization rate of *H* = 17%, the number of new infections and subsequent newly hospitalized patients were exemplarily calculated for *R* = 2. The daily growth rate of new infections was thus found to equal *g*_*eff*_ = 1.189 (Figure 3). With the increasing number of new infections, $i_{100,000}^{7\;days}$ reached and crossed the threshold of $i_{100,000}^{7\;days}=50$ on day 9 (*k* = 9). Assuming social distancing measures were taken, a successive slow reduction of the growth rate ($g_{eff}^{steps}=-0.027$) until *R*_*low*_ = 0.8 (Δ*g*_*eff*_ = −0.2435) could be observed. The maximum number of daily infections amounted up to *I*_max_ = 122 and was observed on day 15 ($t_{peak}^{I}=15$, Figure 3). Mitigation measures were assumed to stay in place for at least *m* = 28 days resulting in a continuous decrease of the number of daily new infections for *k* > 15. Based on the calculated notional number of new infections, Equations (2, 3) were used for the calculation of expected occupancy in NOR and ICU due to a notional number of new COVID-19 patients. The expected occupancy for NOR shows a peak occupancy of 155.1 used beds at $t_{peak}^{NOR}=22$. For ICU, the peak occupancy of 44.8 was reached slightly later at $t_{peak}^{ICU}=25$ (Figure 3).

After 60 days, daily incidences prevailed on a low level (*I* = 10.8), while NOR and ICU occupancy (27.6 on NOR; 16.0 on ICU) were still found to be increased.

**Figure 4:**
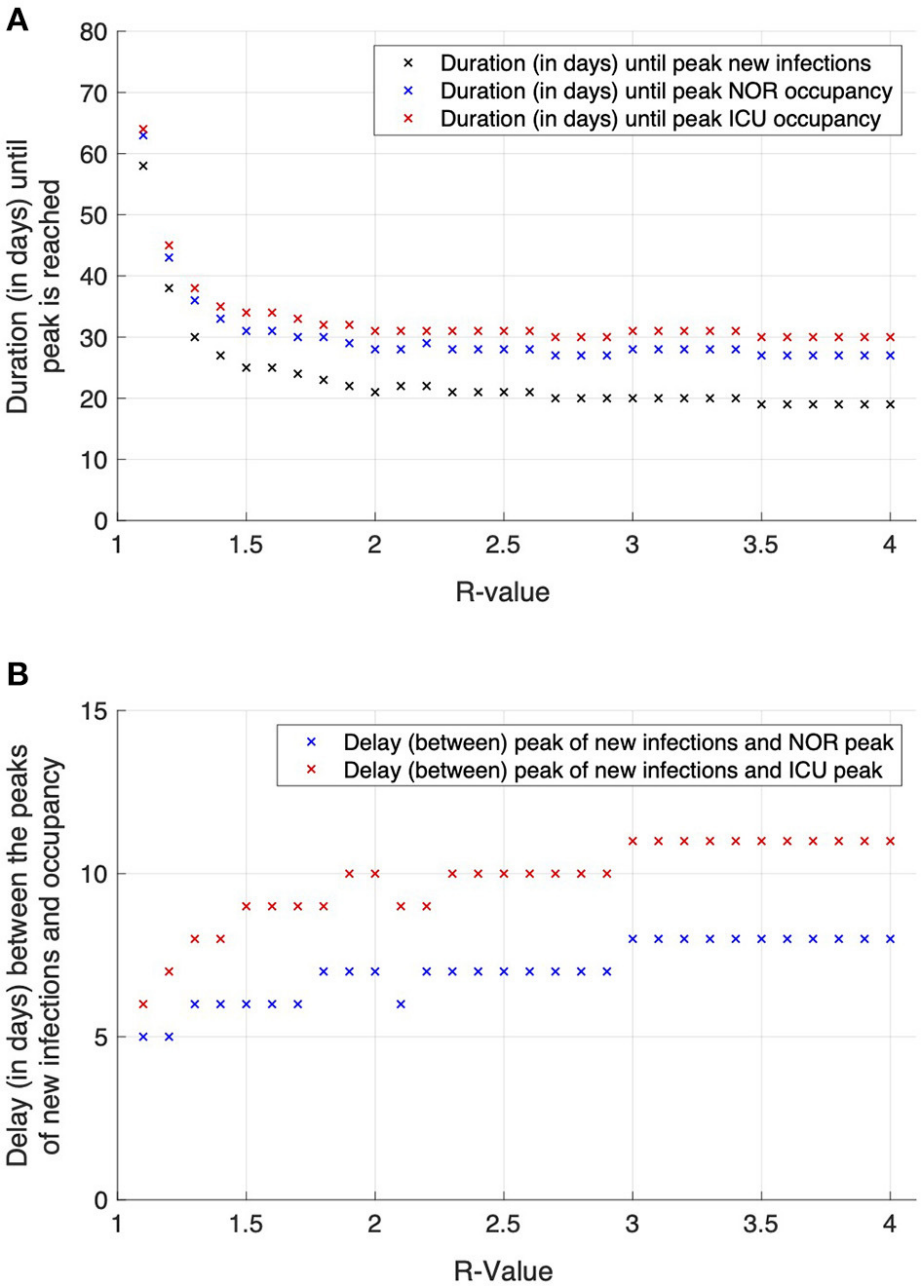
Effects of varying *R*-values on the time to peak for new infections and NOR and ICU occupancy **(A)** and delay between these two measures **(B)**. The higher the *R*-values, the earlier the peaks in the course of the simulation occur **(A)**. Contrastingly, the delay in time between the occurrence of the infection peak and the occupancy peaks for the NOR (red marks) and ICU (blue marks) enlarges with increasing *R*-value **(B)**.

For sensitivity analysis, simulations were repeated with different *R*-values ranging from 1.1 to 4.0 allowing to determine the number of new admissions and their expected occupancy for NOR and ICU, namely the day of maximal number of new infections ($t_{peak}^{I}$), maximal number of new infections (*I*_max_), day and amount of maximal number of NOR occupancy ($t_{peak}^{NOR}$), ($O_{\max}^{NOR}$), and day and amount of maximal number of ICU occupancy ($t_{peak}^{ICU}$), ($O_{\max}^{ICU}$). From these values, the time from maximal daily infections to the day of maximal NOR and ICU occupancy, i.e., $t_{peak}^{NOR}-t_{peak}^{I}$ and $t_{peak}^{ICU}-t_{peak}^{I}$, where calculated together with the relative additional increase after $t_{peak}^{I}$ expressed as factor *a*^*w*^, where $a^{W}=O_{\max}^{w}/O_{t_{peak}^{I}}^{w}$ and *w*= NOR or ICU. The number of days from maximal daily infections to the maximal NOR and ICU occupancy increases with rising R from 5 to 8 (6 to 11) for NOR (ICU), respectively. At the same time, the additional, relative increase *a*^*NOR*^ and *a*^*ICU*^ increases with rising *R* from 1.1 to 1.5 for NOR and from 1.1 to 1.7 for ICU (Figure 4; Table 2). For example, for an *R*-value of 2 the peak in new infections was observed on day 21, while the peak of NOR (ICU) occupancy was observed on day 28 (31). The difference between these days is shown in panel B, namely 7 days for NOR and 10 days for ICU.

**Table 2:** Effects of different *R*-values on the characteristics of the expected occupancy probability.

**Effective reproductions rate** **(R)**	**Days from of peak for new infections to maximal NOR occupancy** ($t_{peak}^{NOR}-t_{peak}^{I}$)	**Relative increase of NOR occupancy after peak of new infection**s ($O_{max}^{NOR}/O_{t_{peak}^{I}}^{NOR}$)	**Days from of peak for new infections to maximal ICU occupancy** ($t_{peak}^{ICU}-t_{peak}^{I}$)	**Relative increase of ICU occupancy after peak of new infections** ($O_{max}^{ICU}/O_{t_{peak}^{I}}^{ICU}$)
1.1	5	1.1	6	1.1
1.5	6	1.2	9	1.3
2.0	7	1.3	10	1.5
2.5	7	1.3	10	1.4
3.0	8	1.4	11	1.5
3.5	8	1.5	11	1.6
4.0	8	1.5	11	1.7

*Several key numbers of the expected occupancy were derived from the following numbers: day of maximal number of new infections ($t_{peak}^{I}$), maximal number of new infections (I_max_), day and amount of maximal number of normal ward (NOR) occupancy ($t_{peak}^{NOR}$), ($O_{\max}^{NOR}$) and day and amount of maximal number of intensive care unit (ICU) occupancy ($t_{peak}^{ICU}$), ($O_{\max}^{ICU}$). From these values, the time from maximal daily infections to the day of maximal NOR and ICU occupancy, i.e., $t_{peak}^{NOR}-t_{peak}^{I}$ and $t_{peak}^{ICU}-t_{peak}^{I}$, was calculated and listed together with the corresponding relative increase of NOR and ICU occupancy after peak of new infections ($O_{\max}^{NOR}/O_{t_{peak}^{I}}^{NOR}$) and ($O_{\max}^{ICU}/O_{t_{peak}^{I}}^{ICU}$)*.

## Discussion

### Initial Data and Hospitalizations Rates

Using data from the first wave of COVID-19 infections between March and May 2020, we modeled *N* = 334 cases from five hospitals in four adjacent counties. The probability of occupancy for NOR and ICU showed an exponential decline (Figure 1), with an ICU starting value equal to 0.27. This means that 27% of hospitalized patients were treated on the ICU for several days (≥1 days), which is slightly higher than found by Nachtigall et al. (21) who reported 0.21 in a German retrospective analysis. These figures surpass prior estimations stating that 3–10% of patients are in need of ICU care (15).

With increasing knowledge about management of covid-19 cases, and especially with the establishment of clinical indicators for the admission to ICUs, admission rates to the ICU were expected to decline. The clinical criteria were hypoxemia with an oxygen saturation lower than 90% with 2–4 L of oxygen supplementation per minute and dyspnea or a respiratory rate higher than 25/min. Furthermore, the routine administration of dexamethasone in patients with type 1 respiratory failure was found to reduce the probability of a severe course of disease (22, 23).

Comparing NOR and ICU average length of stay, the slower decline observed for the ICU indicates that patients treated on ICU stayed longer compared to NOR. On NOR, less than 10% stayed for 20 days and longer. Approximately 1% of patients stayed for 30 days and longer. On the ICU, 10% of all patients (42% of ICU patients) stayed 13 days and longer on the ICU. Only 1% of all subjects (4.3% of ICU patients) stayed 45 days and longer. This is in line with an early German model that estimated an average ICU stay of 14 days (15).

After 60 days, 16 ICU beds were still occupied, which could lead to a potential shortage of beds in a following wave. If several waves followed each other shortly (e.g., <30 days interval), resources could become scare, contradicting Stang et al. (15). Therefore, a rather slow and considered change of mitigation measures is useful and recommended.

The estimated LOS could also be related to bed occupancy rates and the respective epidemiological situation.

For that, we had to make several assumptions. We presumed the incidence of SARS-CoV-2 infections in the area to be equal to the rate in mid-August (17), and the hospitalization rate of 17%, similarly to the first wave in Germany (20).

Hospitalization rates varied largely by country and region. For example, it amounted up to 26% in Belgium (24), 30% in the Netherlands (25), and 69% in France (26). A large-scale international study showed an average hospitalization rate of 6.6% (27). In Germany, it was primarily estimated to lay between 3 and, respectively, 5–10% (15, 28) for the first wave of COVID-19.

It could be argued that in a potential second wave, the hospitalization rate will be subjacent since it was unclear at the beginning of the pandemic whether an outpatient setting was at all feasible for this disease. In this analysis, the hospitalization rate is an important linear constant, meaning that with a 50% lower rate, bed occupancy rates would also halve. However, the shape of the determined curves and thus the course will be left unchanged.

### Influence of Selected *R*-Value and Implementation of Mitigation Measures

Another important assumption was *R*. The effective reproduction rate *R* indicates the average number of people infected by an infectious person if immunity prevails in the population. The number of new infections and subsequently newly hospitalized patients were exemplarily calculated for *R* = 2, resulting in a daily growth rate of new infections of 1.189 (Figure 3). The peak occupancy was expected to be reached with ~155 used beds on NOR at day 22 and with ~45 beds on ICU at day 25.

This simulation was based on effective mitigation measures that were launched on day 9 for at least 28 days. The end of these mitigation measures was conditional to incidences having surpassed 50 per 100,000. The number of days from maximal daily infections to the maximal NOR and ICU occupancy increases with rising *R* from 5 to 8 days (6 to 11 days) for NOR (ICU).

Similar delays from maximal daily infections of the maximal NOR and ICU occupancy were observed in the corresponding data of the first wave in the respective area. These results might indicate that with increasing *R*, the time to provide additional ICU capacities enlarges as well. Since a higher *R* denotes a more critical situation with soaring infection rates, a longer time span for the preparation of hospital staff and medical equipment (e.g., ventilators and beds) could lead to an optimal medical care situation. These five additional days of preparation, i.e., as observed for *R* = 1.1 compared to *R* = 4, might represent a game-changer for the handling of the situation.

Of course, with rising infection rates, the surplus of additionally needed ICU (and NOR) beds increases as well. In this model calculation the demand for ICU beds only slightly exceeds the number of maximum ICU beds from the first wave. However, during the first wave, there was no established limit of the 7-day incidence equal to 50 per 100,000 in place at the level of individual counties. It could be argued that during 1st wave several measures (e.g., social distancing, general lockdown) were taken at stages with different local infection rates, and measures remained in force for a longer time.

**Figure 5:**
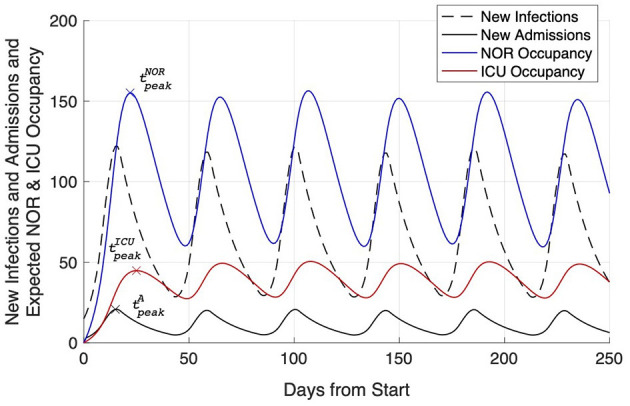
Simulation of the effects of mitigation measures affecting the current *R*-value: new infections (dashed black line), new hospitalizations (solid black line), and expected NOR (blue line) and ICU (red line) occupancy caused by short term measures, i.e., in this case measures that were stopped already 5 days after the 7-days incidence returned below the level of 50.

In the light of recent political discussions in Germany and a new tendency for short term measures, the question arises how bed occupancy rates would consequently develop. With *R* = 2, minimum duration of measures of 5 days and incidence below critical levels, our estimated bed occupancy rates would undulate on NOR and ICU at a higher level (on average 114 for NOR and 42 for ICU, with maximal values of 155 for NOR and 45 for ICU) with an undulation period of 42 days (Figure 5).

This could be discussed as a useful procedure if herd immunity should be approached and existing capacities should be utilized as much as possible (29).

Thus, the following practical implications can be derived:

1. A higher *R* leads to higher bed occupancy rates, but it might take longer to reach the peak occupancy. This provides extra preparation time. In our model, with an *R* = 1.1, the peak was reached 5 days (NOR) and 6 days (ICU) after the peak of infections, with *R* = 4, the peak was reached 8 days (NOR) and 11 days (ICU) after the peak of infections.
2. With onset of measures, hospitals should expect a continued increase of NOR and ICU occupancy (typical factors are 1.1–1.7 depending on *R*), therefore hospitals should further reduce standard care in favor of COVID-19 patients. Hospitals have about 5–11 days before the maximum bed occupancy might be expectantly reached.
3. With political mitigation measures taken for short periods of time at similar or lower 7-day incidences compared to the first wave, a clearly definable next wave might be avoided. Our model predicts in this case undulations in bed occupancy rates with peaking occupancy approximately every 6 weeks depending on *R* and duration of the measures taken. However, this would impose even greater challenges on clinicians balancing routine and COVID-19 care.

Of note, all actions of the civil protection and hospital management units should primarily be tuned and scheduled on the dynamics of the occurrence of infections, reflected by the *R*-value, and the threshold of available beds on the different wards (NOR, ICU). The IVENA database (ILS FFB IVENA eHealth), i.e., a web based program where all hospitals have to update their NOR and ICU occupancy and the maximal available number of beds on the different wards allows the level of occupancy to be tracked in real time to control the availability of resources. The maximum number of available beds is, however, not corresponding to the actual physical beds, but foremost on the available health care workers. These human resources have been often considered as “the bottleneck” as they are finite and not allocatable according to the outbreaks' pressure.

In a second step and after these mandatory control mechanisms are in place, it is time to plan the emergency preparedness against the maximum value of NOR and ICU beds. The model results indicate that high initial *R*-values might be associated with slightly larger delays between the peak of new infections and the peak occupancy on NOR and ICU. However, planning should be done with the minimally observed delays, which inform about the availability to set up additional resources, including additional beds, devices, and staff.

The observed increased delay in time to peak occupancy rates with increasing *R* follow the subsequent rationality: Given a constant incidence, occupancy rates on both, NOR and ICU, will reach a constant value as well (only COVID-19 cases taken into account). The curves for the likelihood of occupancy (Figure 2) define the inertia, i.e., the time until a constant NOR and ICU occupancy rate is reached. For example, independent of the constant incidence, it takes 20 days for NOR and 40 days for ICU to reach a constant level, i.e., 95% of the maximal level. Second, the *R*-value controls the skewness of the incidence. For higher *R*-values, the exponential increase is sharper as compared to lower *R*-values close to 1. Therefore, for higher *R*-values, due the inertia, NOR and ICU occupancy rates increase as fast as the incidence, resulting in a slight shift toward longer delays of time to peak for larger *R*-values.

This model, however, does not consider the period from detection of infection to hospitalization.

It is important to note that it typically takes several days from the detection of infection to hospital admission. Such a delay would be expected to lead to a prolonged period between the incidence and the occupancy rates summits. This could reduce the criticality of emergency resource allocation slightly. This delay was not considered in this model, which can be seen as a limitation of this model.

### Validation of Model With Data From the 2nd and 3rd Waves

**Figure 6:**
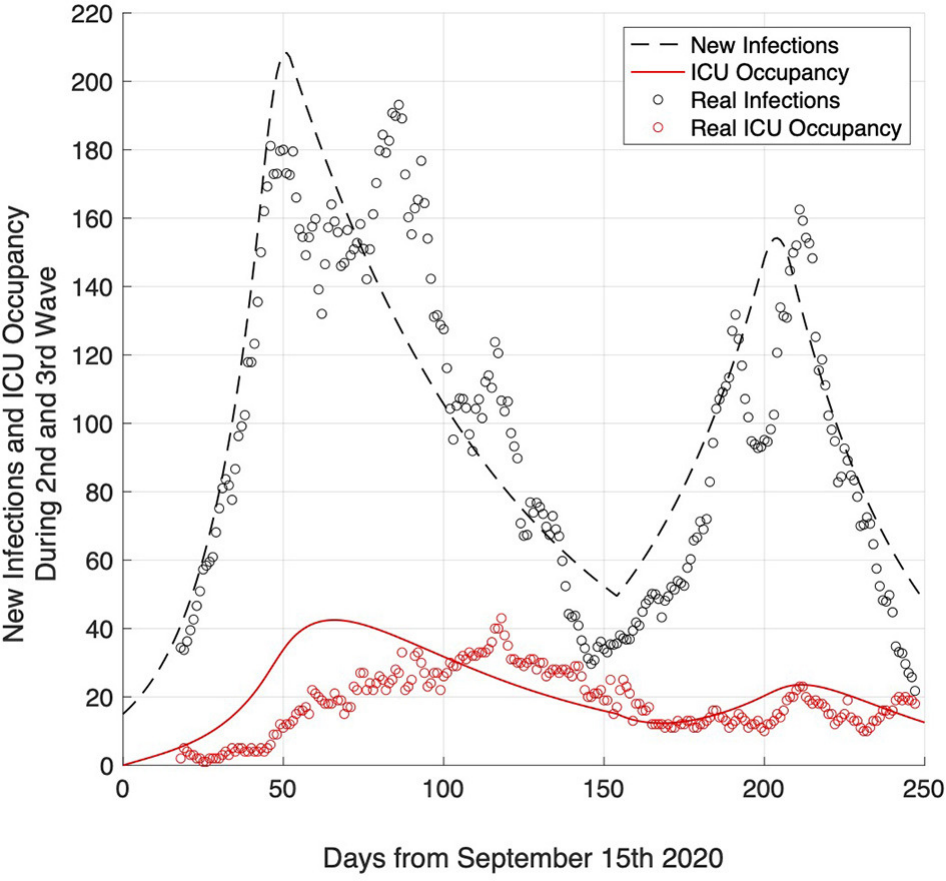
Validation of the model by comparing simulated number of infection rates (dashed black line) and ICU occupancy (red line) with real data of infection rates (black circles) and ICU occupancy (red circles) obtained during the 2nd and 3rd wave (September 2020–May 2021) in the four counties south-west of Munich (ZRF FFB).

For validation purposes, the presented model was applied to the respective pandemic situation in the four counties south-west of Munich during the 2nd and 3rd waves (Figure 6). Data covers 250 days from September 15th 2020 to May 26th 2021. New infections (data source: RKI) are represented by black circles (Figure 6, slighting windows of 7 days). ICU occupancy rates (data source: ILS FFB IVENA eHealth) are represented by red circles (Figure 6). Because of a change of pandemic dynamics over time, simulation parameters required several adaptations: for the 2nd wave, *R* = 1.25 and *R*_min_ = 0.75 were used to describe the obtained date. Furthermore, the decline in the incidence due to mitigation measures (starting at a threshold of 160 for 75 days) was done in 16 steps to cope with the observed prolonged peaks of the new infections. For the 3rd wave, *R* = 1.1 and *R*_min_ = 0.8 were assumed as they best described the 3rd wave and likewise, the reduction due to mitigation measures (starting at a threshold of 150 for 60 days) was done in 14 steps. Vaccination programs (with priority in the elderly people) and the increasing fraction of the population recovered from COVID-19 require a consequent reduction of the R-value from wave to wave. The vaccination program started in the four counties south-west of Munich in late December 2020 (Figure 6, day 100). During 2nd and 3rd wave, reduced hospitalization rates to ICU were observed, which were also reflected in the simulation by using hospitalization rates of 7% and 5% for 2nd and 3rd wave simulation, respectively. The decline of ICU bed occupancy rates might be related to the early vaccination of high-risk population, i.e., elderly and people with comorbidities.

The expected number of new infections (Figure 6, dashed black line) and ICU occupancy (Figure 6, red line) constitute a good fit for the real data. The peak in ICU occupancy occurred later than expected, here it might be necessary to consider a further parameter, namely a delay between infection and ICU admission, which might be in the range of 2–3 weeks.

### Model Limitations

Subsuming, this relatively simple model does not take into account several aspects of the epidemiological situation and more complex models are available. Nevertheless, we consider it an adequate and practical basis for hospital leaders to derive the need for COVID-19 care in the light of the respective epidemiological situation. Length of stay probabilities derived from the obtained data could be applied to other models as well.

Although several model parameters like hospitalization rate have been selected based on data from numerous Germany hospitals, other used data represented only four counties south-west of Munich in five hospitals. Further research has to verify whether our findings could also be applied nationwide or even internationally.

Furthermore, political measures currently in discussion can only be reflected in the model when leading to a reduction in *R* below 1. Other factors leading to a limitation of transmission, like herd immunity, have not been regarded for reasons of simplicity.

## Conclusion

Due to Nowcasting of the *R*-value, hospital leaders are now precisely informed about the infection rates in their counties. Our model based on data from four counties south-west of Munich provides a relatively simple framework of how the demand for ICU and NOR beds reacts to certain infection rates and political measures. Furthermore, it provides a solid example of the complex relationships and time spans and reduces uncertainty in the provision of ICU capacities. The main results were the concordant relationship between increased daily infection rates and maximal NOR and ICU occupancy rates, indicating a self-stabilization of supply and demand at a high bed occupancy rate. After a peak in infections, hospitals have about 5–11 days before the maximum bed occupancy is expected to be reached. Depending on the duration of political mitigation measures and *R*, bed occupancy rates might undulate with small peaks approximately every 6 weeks. However, this would impose even greater challenges on clinicians balancing routine and COVID-19 care. Further research should focus on real time modeling of these connections to provide day to day updates.

## Data Availability Statement

The raw data supporting the conclusions of this article will be made available by the authors, without undue reservation.

## Ethics Statement

Ethical review and approval was not required for the study on human participants in accordance with the local legislation and institutional requirements. Written informed consent for participation was not required for this study in accordance with the national legislation and the institutional requirements.

## Author Contributions

FS, CH, and LN drafted the work, revised it critically for important intellectual content, and agreed to be accountable for all aspects of the work in ensuring that questions related to the accuracy or integrity of any part of the work are appropriately investigated and resolved. FS, CH, WK, PG, SH, MD, TW, and LN contributed substantially to the acquisition of data, their analysis, and interpretation of the work. All authors approved the final version for publishing.

## Conflict of Interest

The authors declare that the research was conducted in the absence of any commercial or financial relationships that could be construed as a potential conflict of interest.

## Publisher's Note

All claims expressed in this article are solely those of the authors and do not necessarily represent those of their affiliated organizations, or those of the publisher, the editors and the reviewers. Any product that may be evaluated in this article, or claim that may be made by its manufacturer, is not guaranteed or endorsed by the publisher.
