# Supplementary material for: Tackling the Waves of COVID-19: A Planning Model for Intrahospital Resource Allocation
Source: Front Health Serv. 2021 Nov 16;1:718668. doi: 10.3389/frhs.2021.718668 (PMC10012637; doi:10.3389/frhs.2021.718668)
Supplement: Supplementary file 1 [file Data_Sheet_1.PDF]

Online Supplement

Supplemental Tables

SUPPLEMENTAL TABLE 1: Hospitals

	Number of hospital beds	Maximal number of ICU beds	Medical Specialties	County	Population
Gauting	250	35	Internal medicine (pulmonary and bronchial medicine. including related thoracic and skeletal surgery for tuberculosis)	Starnberg	135.910
Starnberg	308	8	Surgery. gynecology. Otorhinolaryngology. internal medicine. pediatrics. neurology. urology. hemodialysis		
Dachau	435	58	Surgery. gynecology. Otorhinolaryngology. internal medicine. neurology. urology	Dachau	154.544
Fürstenfeldbruck	380	15	Surgery. gynecology. Otorhinolaryngology. internal medicine. urology	Fürstenfeldbruck	219.382
Landsberg	218	10	Surgery. gynecology. Otorhinolaryngology. internal medicine. pediatrics	Landsberg	120.089
Total	1.591	126			629.925

Note: from "Bayerischer Krankenhausplan 2020"; Max ICU Information from ICENA (June 16th, 2020); population information from county websites

METHODOLOGICAL SUPPLEMENT.

Calculation of an effective daily growth rate: The daily amount of new infections was calculated using an effective daily growth rate (g_{eff}) derived from the assumed R-value. This means that the number of new infections were calculated based on infections of the previous day and the effective growth rate of the respective day. This is a valid approach for the estimation of the actual *R*-value, since for new emerging infectious diseases, like the COVID-19 pandemic, this number is yet to be determined. There are different equations which related the *R*-value to the growth rate of infections and in new infectious diseases, it is yet to be determined, which to apply. Following Wallinga & Lipsitch (2007), these equations differ mostly regarding assumed shape of the generation interval distribution. When the generation interval distribution is set equal to the observed distribution, it is possible to obtain an empirical estimate of the reproductive number. Following an der Heiden & Hamouda (2020), the *R*-value is the quotient of the number of new cases in two successive periods of 4 days, given a constant generation time of 4 days, as observed in Germany in April 2020. If the number of new cases has increased in the second period, *R* is above 1. If the number of new cases is the same in both periods, the reproduction number equals 1. This then corresponds to a linear increase in the number of cases. If, on the other hand, only every second case infects another person, i.e. *R* = 0.5, then the number of new infections is halved within the generation time. In our model, the daily growth rate and R-value are linked by $g = \sqrt[4]{R}$, since two subsiding four-day intervals are used to calculate the R-value.
